# An annotated species list of regular echinoids from Sri Lanka with notes on some rarely seen temnopleurids

**DOI:** 10.11646/zootaxa.4571.1.3

**Published:** 2019-03-25

**Authors:** Gayashan M. Arachchige, Sevvandi Jayakody, Rich Mooi, Andreas Kroh

**Affiliations:** 1 Department of Aquaculture and Fisheries, Wayamba University of Sri Lanka, Makandura, Gonawila, Sri Lanka; 2 Department of Invertebrate Zoology and Geology, California Academy of Sciences, 55 Music Concourse Drive, San Francisco, CA 94118, USA; 3 Natural History Museum Vienna, Department of Geology and Palaeontology, Burgring 7, 1010, Vienna, Austria

**Keywords:** Echinoidea, species list, fauna, biodiversity, Sri Lanka

## Abstract

The first recorded regular echinoid species from Sri Lanka, “*Salmacis virgulatus*” (now known as *S. virgulata* L. Agassiz in L. Agassiz & Desor, 1846), was listed by Agassiz & Desor (1846). Knowledge of Sri Lankan regular echinoids continued to advance until the end of the 19^th^ century. However, there is a gap in knowledge between the mid-20^th^ and the beginning of the 21^st^ centuries due to a lack of systematic studies, with the exception of two checklists published by the IUCN Red List in 2006 and 2012. In the present study, we compiled a species list combining published data and new data based on fieldwork between 2013 and 2015. Echinoids were sampled by snorkelling, diving, examination of fisheries bycatch, and collection of tests from beaches. The updated species list presented in this study includes 39 regular echinoids belonging to 28 genera, nine families, and five orders. *Phyllacanthus imperialis* (Lamarck, 1816) and *Temnopleurus toreumaticus* (Leske, 1778), which were not recorded during the last 90 years, were confirmed to still occur in Sri Lanka by the present study. We develop an updated species list of regular echinoids to form a basis for future systematic work. The study recommends further investigation to evaluate the status of deep-sea species and additional field work off the northern and eastern coasts of Sri Lanka.

## Introduction

The Echinoidea includes more than 1,000 living species in 70 families (Kroh & Smith 2010; Appeltans *et al.* 2012), and can be divided into two groups, so-called “regular” and irregular, by considering their gross morphology. Only the latter group is monophyletic. In contrast to irregular echinoids, which are bilaterally symmetric and have the periproct (containing the anus) at the functionally posterior part of the body, the regular echinoids have a pentaradially symmetric body, or test (Serafy & Fell 1985). In these forms, the periproct is within the apical system at the top (aboral surface) of the animal, opposite the mouth situated on the bottom (oral surface) (Mortensen 1928; Durham & Wagner 1966; Melville & Durham 1966; Saucède *et al.* 2007). The regular echinoids constitute a paraphyletic assemblage (Smith & Kroh 2011) that includes slate pencil urchins (cidaroids), soft-bodied fire urchins (echinothurioids), and other sea urchins (diadematoids, camarodonts, and other carinacean forms) with large spines and a subspherical body (Mooi & Munguia 2014). Regular echinoids are considered keystone epibenthic organisms in rocky substrates and reefs (Barnes *et al.* 2002; Cebrian & Uriz 2006) as well as in soft bottom habitats (Hardy *et al.* 2011).

“*Salmacis virgulatus*” (now known as *S. virgulata* L. Agassiz in L. Agassiz & Desor, 1846) was the first recorded member of the regular echinoid fauna of Sri Lanka. After this publication, several authors reported many regular echinoids from Sri Lankan waters during the early 19^th^ and mid-20^th^ centuries (Agassiz 1872–1874; Bell 1882; Walter 1885; Sarasin, 1888; Sarasin & Sarasin 1886, 1887, 1888; Döderlein 1888; Herdman *et al.* 1904; Clark 1907, 1915, 1917, 1925; Mortensen 1928, 1935, 1940, 1943a, 1943b; Clark & Rowe 1971). This was followed by only sporadically published records for several decades, creating a gap in knowledge of these echinoids between 1928 and 2006 (see Arachchige *et al.* 2017 for review). Work carried out by Malik Fernando, Prassana Weerakkody, Sevvandi Jayakody, Gayani Thilakaratne, and Gayashan M. Arachchige helped increase the knowledge of echinoid diversity in Sri Lanka (Jayakody 2012), and confirmed the presence of some species recorded by previous authors.

The present study aims to expand the knowledge about the species richness of regular echinoids in Sri Lankan waters. The results of this research, which combines data collected between 2013 and 2015 with that from existing publications, provide an updated, annotated species list of Sri Lankan regular echinoids.

## Materials & methods

**Figure 1:**
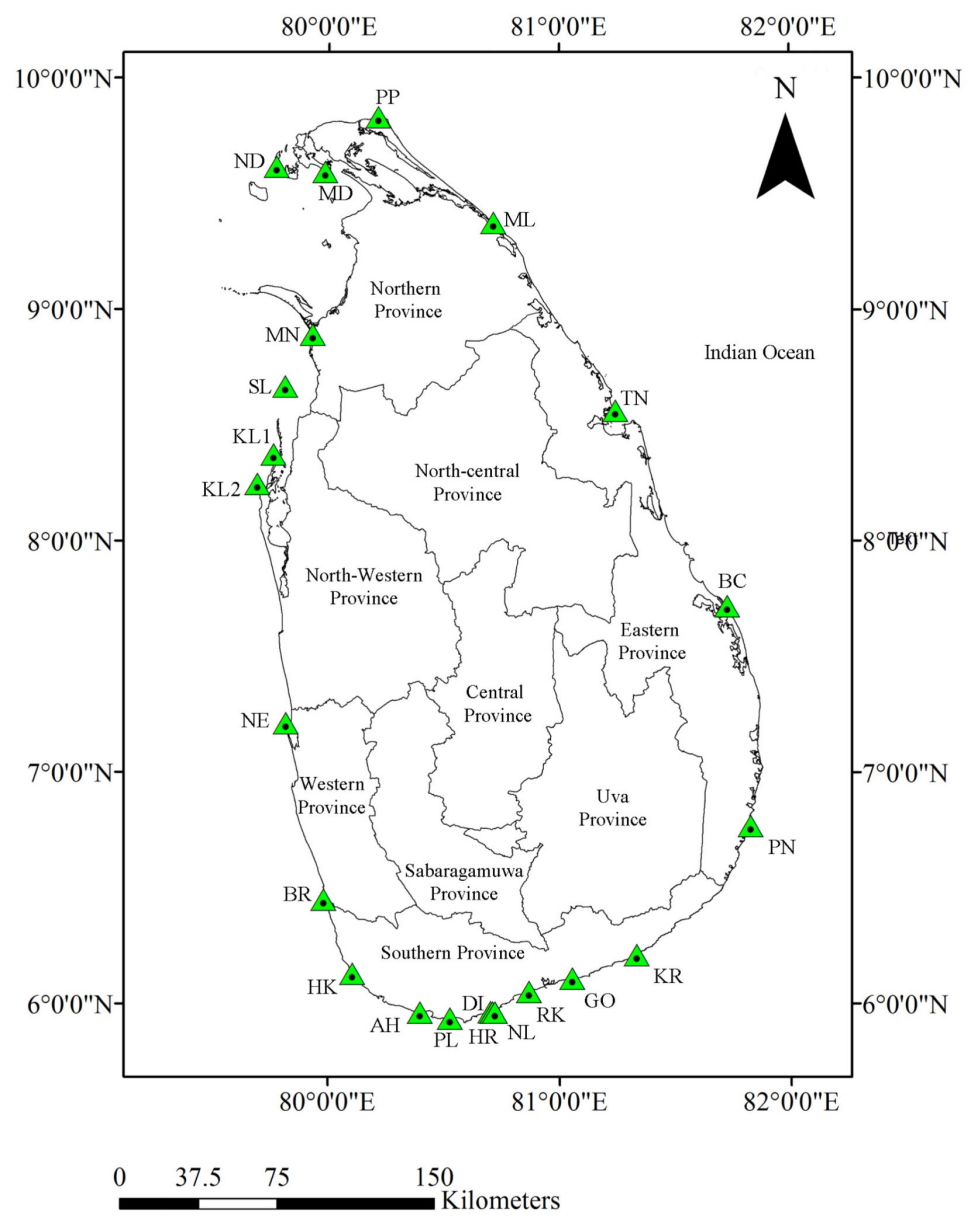
Map of sample collection and observation sites (AH—Ahangama, BC—Batticaloa, BR—Beruwala, DI—Dickwella, GO—Godawaya, HK—Hikkaduwa, HR—Hiriketiya, KL1—Kalpitiya 1, KL2—Kalpitiya 2, KR—Kirinda, MD—Mandathiv, MN—Mannar, ML—Mulathiv, ND—Nagadeepa, NE—Negombo, NL—Nilwella, PN—Panama, PP—Point Pedro, PL—Polhena, RK—Rakawa, SL—Silavathurai, TN—Trincomalee; see Appendix 1 for more information).

Echinoid specimens were collected between January 2013 and January 2015 from 22 localities along the Sri Lankan coastline (Fig. 1) by snorkelling and SCUBA diving (up to 33 m depth). Dead, beach-washed specimens were gathered at low tide on the shore and from among discarded specimens at fish landing sites. Fresh specimens were preserved in 10% formalin (indicated by “wet” in the material lists below).

All the newly collected specimens are deposited in the Department of Aquaculture and Fisheries (DAF) of Wayamba University of Sri Lanka (WUSL). Details of sites from which species were recorded during the field work are given in Appendix 1. Irregular echinoids collected during the field work were described by Arachchige *et al.* (2019).

In addition to the data from the present research, published literature, species deposited in the DAF, WUSL, and records of Sri Lankan samples registered in the Invertebrate Zoology collection database of the California Academy of Sciences, San Francisco (CAS) (https://www.calacademy.org/scientists/izg-collections) were used for the compilation of the species list of “regular” echinoids of Sri Lanka. The specimens at DAF were collected by R.M.G.N. Thilakaratne and S. Jayakody during 2007–2008. Locality information extracted from published literature is given in Appendix 2.

Identification of specimens was based on the keys developed by Mortensen (1928, 1935, 1940, 1943a, 1943b), Clark & Rowe (1971), Schultz (2005, 2011), and on the Echinoid Directory, which is an online key hosted by the Natural History Museum, UK (Smith & Kroh 2011). In addition, we included species that we were unable to examine but which were included in publications with references to Sri Lanka, to create the updated species list. The taxonomic list and nomenclature were organised and updated systematically according to the World Echinoidea Database (Kroh & Mooi 2018).

## Results

Fifteen species were recorded and identified during the fieldwork, representing 12 genera, six families, and four orders (Table 1). In addition, two species, *Astropyga radiata* and *Pseudoboletia maculata*, were found in the DAF collection. Apart from that, 22 regular echinoid species were added to this species list based on the most recent literature compilation done by Arachchige *et al.* (2017). Thus, a total of 39 regular echinoid species belonging to 28 genera, nine families, and five orders are included in this species list (Table 1). Two species, *Phyllacanthus imperialis* and *Temnopleurus toreumaticus,* are recorded for the first time since they were last mentioned in a scientific publication on Sri Lankan echinoids 90 years ago. The following descriptions report only the new material collected during our fieldwork, plus the two DAF specimens. For details on species records solely based on literature records and not resampled during the present study, see Arachchige *et al.* (2017). Here, for the first time, we provide photographs of hitherto unillustrated test surface details of poorly known, and seldom encountered temnopleurids now known to occur in Sri Lanka.

**Systematic part**

**Class Echinoidea Leske, 1778**

**Subclass Cidaroidea Smith, 1984**

**Order Cidaroida Claus, 1880**

**Family Cidaridae Gray, 1825**

***Phyllacanthus imperialis* (Lamarck, 1816)**

**Material studied.** WUSL/ER/198, 199 (wet, with spines) from Godawaya; WUSL/ER/200 (dry, with spines) from Negombo; WUSL/ER/201, 202 (dry, denuded) from Nilwella.

**Literature records for Sri Lanka.** Agassiz (1872), Herdman *et al.* (1904), Clark (1915, 1925).

**Distribution in Sri Lanka.** Southern and western coasts of Sri Lanka.

**Recorded depth range in Sri Lanka.** 1–8 m (present study), 23–65 m (previous records).

**Habitat.** In coral ecosystems where it hides during the day in rock beds and emerges at night to graze.

**Observed occurrence in this study.** Southern coast (Godawaya, Nilwella) and western coast (Negombo) of Sri Lanka.

**Remarks.** This species differs from others in the genus by its characteristic primary spines with very numerous, closely spaced series of granules. Mortensen (1928) introduced three forms under *Phyllacanthus imperialis*: *typicus*, *ustigerus*, and *unicolor*. Specimens observed during this study most closely matched *unicolor.* Their primary spines are unbanded, and uniformly dark violet. This species was recorded from Sri Lanka 90 years ago by Clark (1925).

**Order Diadematoida Duncan, 1889**

**Family Diadematidae Gray, 1855**

***Astropyga radiata* (Leske, 1778)**

**Material studied.** WUSL/ER/203 (dry; denuded) from Trincomalee.

**Literature records for Sri Lanka.** Sarasin & Sarasin (1887), Döderlein (1888), Anderson (1894), Fernando (2006), Jayakody (2012).

**Distribution in Sri Lanka.** Eastern coast of Sri Lanka.

**Recorded depth range in Sri Lanka.** 51 m (Anderson 1894).

**Habitat.** Not recorded.

**Observed occurrence in this study.** None.

**Remarks.** This species differs from other diadematoids found in Sri Lanka by having a very low test, conspicuously elongated, narrow genital plates, and spines in which the shaft is partially filled with a loose stereom meshwork.

Sarasin & Sarasin (1887), Döderlein (1888), and Anderson (1894) recorded this species from Trincomalee. One denuded specimen is housed at the Department of Aquaculture and Fisheries of the Wayamba University of Sri Lanka. This specimen was collected from off Trincomalee, but its depth is unknown.

***Diadema savignyi* (Audouin, 1809)**

**Material studied.** WUSL/ER/204 (dry, denuded) from Nilwella; WUSL/ER/209 (dry, denuded) from Hiriketiya.

**Literature records for Sri Lanka.** Clark (1915), Fernando (2006), Jayakody (2012), Gayashan & Jayakody (2012).

**Distribution in Sri Lanka.** Southern coast of Sri Lanka.

**Recorded depth range in Sri Lanka.** 0.5–4 m (present study).

**Habitat.** Shallow reef areas.

**Observed occurrence in this study.** Southern coast (Hiriketiya and Nilwella) of Sri Lanka.

**Remarks.** The test of adult *D. savignyi* can be distinguished from that of adult *D. setosum* because the former has up to five primary tubercles in one horizontal series in the interambulacra, and the latter has six to seven. In life, *D. savignyi* lacks a red ring around the anus, but has bright blue lines around the plates in the apical system, and two paired, thin lines of the same vivid color running down along each interradial suture towards a white, iridescent spot. At this point, the two blue lines diverge to run along the center of each column of interambulacral plates. In *D. savignyi*, right blue lines can often be seen around the bases of the primary spines.

Clark (1915) reported this species in Sri Lanka for the first time based on two specimens housed at the Colombo Museum. However, no exact locality nor depth records are available. Clark’s collection is no longer available at the Colombo Museum.

***Diadema setosum* (Leske, 1778)**

**Material studied.** WUSL/ER/208 (dry, denuded) from Polhena.

**Literature records for Sri Lanka.** Bell (1882, 1887), Sarasin & Sarasin (1887), Döderlein (1888), Herdman *et al.* (1904), Clark (1925), Price & Rowe (1996), Fernando (2006), Jayakody (2012), Gayashan & Jayakody (2012).

**Distribution in Sri Lanka.** Eastern and southern coasts of Sri Lanka.

**Recorded depth range in Sri Lanka.** 0.5–3 m (present study), 0–62 m (previous records).

**Habitat.** Coral reefs, rocky reef platforms.

**Observed occurrence in this study.** Southern coast (Hiriketiya, Nilwella, and Polhena) of Sri Lanka.

**Remarks.** Adults of this species differ from *D. savignyi* by having six to seven primary tubercles in one horizontal series in the interambulacra, a blue spot on each genital plate, and white spots along the interradii instead of lines. In addition, there is a prominent red ring around the anus at the tip of the inflated anal sac.

This species has been recorded consistently from the southern and eastern coasts of Sri Lanka since it was first mentioned as part of the Sri Lankan echinoid fauna (Bell 1882). According to Lessios *et al.* (2001), *D. setosum* contains two mitochondrial lineages that split 3 to 5 million years ago and are now geographically separated. No specimens from Sri Lanka were included in the study by Lessios and co-workers, but species distribution modelling by Bronstein *et al.* (2017) predicts that the Sri Lankan representatives belong to *D. setosum* clade a. Genetic analyses are needed to confirm the prediction based on modelling.

***Echinothrix calamaris* (Pallas, 1774)**

**Material studied.** WUSL/ER/210 (dry, with spines) from Nilwella; WUSL/ER/211 (dry, denuded) from Polhena.

**Literature records for Sri Lanka.** Jayakody (2012).

**Distribution in Sri Lanka.** Southern coast of Sri Lanka.

**Recorded depth range in Sri Lanka.** 1–2 m (present study).

**Habitat.** Reef flats and tide pools.

**Observed occurrence in this study.** Southern coast (Hiriketiya, Nilwella, and Polhena) of Sri Lanka.

**Remarks.** The test of *E. calamaris* can be distinguished from that of *E. diadema* by having naked adapical medial zones in the interambulacra, conspicuously inflated aboral ambulacra, no enlarged ambulacral tubercles at the ambitus and small auricles with low connecting ridges in the interambulacra. In life, *E. calamaris* is easily distinguished from *E. diadema* in having banded, usually pale or white primary spines, and concentrations of gold or light brown, poison-gland bearing spines in the ambulacra that are much shorter and more sharply pointed than the interambulacral primaries. See Coppard & Campbell (2006) for an in-depth discussion of the test features distinguishing the two species.

This species was recently added to the Sri Lankan echinoid faunal list by Jayakody (2012). Our study confirms the presence of *E. calamaris* in Sri Lanka. *E. calamaris* is widely distributed from the western Indian Ocean (Samyn 2003) to the eastern Indian Ocean (Putchakarn & Sonchaeng 2004; Sastry 2007) and beyond (see Clark & Rowe 1971 for a summary).

***Echinothrix diadema* (Linnaeus, 1758)**

**Material studied.** WUSL/ER/212 (wet, with spines) from Hikkaduwa; WUSL/ER/213 (dry, denuded) from Hikkaduwa; WUSL/ER/214 (dry, denuded) from Hiriketiya; WUSL/ER/215 (dry, with spines) from Nilwella; WUSL/ER/216 (wet, with spines) from Nilwella.

**Literature records for Sri Lanka.** Herdman *et al.* (1904), Clark (1915), Fernando (2006), Jayakody (2012).

**Distribution in Sri Lanka.** Southern coast of Sri Lanka.

**Recorded depth range in Sri Lanka.** 1–2 m (present study), 62 m (previous records).

**Habitat.** Coral reefs and rocky reef platforms.

**Observed occurrence in this study.** Southern coast (Hiriketiya, Nilwella, and Hikkaduwa) of Sri Lanka.

**Remarks.** The test of *E. diadema* differs from that of *E. calamaris* in having no naked adapical medial zones in the interambulacra, no inflated aboral ambulacra, enlarged ambulacral tubercles at the ambitus, and large auricles with high connecting ridges in the interambulacra. In life, *E. diadema* tends to be black with bluish iridescence in strong sunlight, and the poison-gland bearing spines are not differentiated in color from the other primary spines.

**Order Stomopneustoida Kroh & Smith, 2010**

**Family Stomopneustidae Mortensen, 1903**

***Stomopneustes variolaris* (Lamarck, 1816)**

**Material studied.** WUSL/ER/217 (wet, with spines) from Nilwella; WUSL/ER/218 (dry, with spines) from Nilwella; WUSL/ER/219 (dry, with spines) from Hiriketiya, WUSL/ER/220 (dry, denuded) from Beruwala; CASIZ 100705, 100778, 101977, 103168 (four wet specimens, with spines), CASIZ 101939 and 102241 (two dry specimens, with spines) all from Ambalangoda.

**Literature records for Sri Lanka.** Walter (1885), Döderlein (1888), Herdman *et al.* (1904), Clark (1915), Koehler (1927), Price & Rowe (1996), Fernando (2006), Jayakody (2012), Gayashan & Jayakody (2012).

**Distribution in Sri Lanka.** All coasts possess suitable habitat for this species.

**Recorded depth range in Sri Lanka.** 0.1–5 m (present study), 5 m (previous records).

**Habitat.** Subtidal rocks, common on rocky platforms, in crevices, under boulders, and in coral reefs; well adapted to areas in which wave action is high.

**Observed occurrence in this study.** Eastern (Batticaloa, Panama, and Trincomalee), northwestern (Kalpitiya 1), southern (Ahangama, Hikkaduwa, Hiriketiya, Kirinda, Nilwella, Polhena, and Rakawa) and western coasts (Beruwala, Negombo) of Sri Lanka.

**Remarks.** The family Stomopneustidae has only one extant species. *S. variolaris* can be distinguished from diadematoids (with some of which it might be confused in life) found in Sri Lanka in having imperforate, non-crenulate primary tubercles, broad multiserial pore zones from the peristomial margin to the apex, large ambulacral tubercles, and a comparatively small apical system that is firmly integrated into the corona (not loosely attached by soft tissues as is usually the case in diadematoids). *S. variolaris* differs from camarodonts in having an open foramen (epiphyses not joined over the teeth in the Aristotle's lantern). Characteristic features of the test include the conspicuously sunken, sinuous interradial sutures and the polygeminate ambulacra with pores that are not aborally arranged in clear arcs.

In Sri Lanka, this species is widely distributed mainly from the southern to the northwestern coasts.

**Order Camarodonta Jackson, 1912**

**Family Echinometridae Gray, 1855**

***Echinometra* ex grupo *mathaei* (Blainville, 1825)**

**Material studied.** WUSL/ER/225 (dry, denuded) from Hiriketiya, WUSL/ER/226 (dry, denuded) from Hikkaduwa; WUSL/ER/227 (wet, with spines) from Beruwala.

**Literature records for Sri Lanka.** Clark (1915, 1925), Price & Rowe (1996), Fernando (2006), Jayakody (2012), Gayashan & Jayakody (2012).

**Distribution in Sri Lanka.** Southern and western coasts of Sri Lanka.

**Recorded depth range in Sri Lanka.** 0.5–1 m (present study), 0.5–5 m (previous records).

**Habitat.** Rocky shores, in rock crevices, among rock boulders, channels, and self-made burrows.

**Observed occurrence in this study.** Southern coast (Hiriketiya and Hikkaduwa) and the western coast (Beruwala) of Sri Lanka.

**Remarks.** *E. mathaei* can be distinguished from the other Sri Lankan echinometrids, except from *E. oblonga*, in having the test elongated through the axis between ambulacrum I and interambulacrum 3, and only four pore pairs in the pore arcs of the ambulacra.

*E. mathaei* and *E. oblonga* cannot be easily distinguished from each other. Mortensen (1943b: 394) admits that “there are no reliable characters in the test distinguishing *oblonga* from the typical *mathaei*”. Hence, molecular analyses are required to distinguish these species unequivocally, although sperm morphology and spicules have been shown to be very useful in distinguishing some members of the *E. mathaei* species complex (Arakaki *et al.* 1998; Bronstein & Loya 2013).

Two colour variants, green and brown, occur in Sri Lanka. There is a high likelihood that more than one species is present on the island, pending full molecular analyses of additional specimens from across the range of echinometrids currently listed under the names *E. mathaei* and *E. oblonga*.

***Echinostrephus molaris* (Blainville, 1825)**

**Material studied.** WUSL/ER/228 (dry, denuded) from Nilwella; WUSL/ER/229 (dry, denuded) from Kalpitiya 2; WUSL/ER/230 (dry, denuded) from Hikkaduwa; WUSL/ER/231 (dry, denuded) from Hiriketiya.

**Literature records for Sri Lanka.** Döderlein (1888), Herdman *et al.* (1904), Clark (1915), Schultz (2005), Fernando (2006), Jayakody (2012), Gayashan & Jayakody (2012).

**Distribution in Sri Lanka.** Northern, southern, and northwestern coasts of Sri Lanka.

**Recorded depth range in Sri Lanka.** 0.5–13 m (present study), 13–24 m (previous records).

**Habitat.** Mostly in burrows in flat, rocky reef bottoms.

**Observed occurrence in this study.** Northwestern (Kalpitiya 2) and southern coast (Hikkaduawa, Hiriketiya, and Nilwella) of Sri Lanka.

**Remarks.** *E. molaris* can be distinguished from the other echinometrid species recorded in Sri Lanka, *Colobocentrotus* (*Podophora*) *atratus*, *Echinometra* spp., and *Heterocentrotus mamillatus*, in having three pore pairs in the pore arcs of the ambulacra and a unique lateral aspect, with the ambitus located high on the test and the aboral side distinctly flattened. *E. molaris* also has a small, circular test with a flattened, broad aboral side. Unique to the genus, the longest spines project vertically in an aboral tuft, whereas the spines on the ambitus and oral surface are extremely short.

This species was first recorded from Sri Lanka by Döderlein (1888) under the incorrectly formed name “*Echinostrephus molare*”.

***Heterocentrotus mamillatus* (Linnaeus, 1758)**

**Material studied.** WUSL/ER/232, 233 (wet, with spines) from Nilwella.

**Literature records for Sri Lanka.** Fernando (2006), Jayakody (2012).

**Distribution in Sri Lanka.** Southern coast of Sri Lanka.

**Recorded depth range in Sri Lanka.** 2–5 m (present study).

**Habitat.** Among rock boulders and in rock crevices.

**Observed occurrence in this study.** Southern coast (Hiriketiya and Nilwella) of Sri Lanka.

**Remarks.** *H. mamillatus* can be distinguished from the other Sri Lankan echinometrids in having the test transversely elongated through the axis between ambulacrum II and interambulacrum 4, and very strongly developed, solid, thick primary spines that are bright red-brown in life. Distally, these spines are almost triangular in cross-section. The secondary spines are extremely short, truncated, and with a flattened tip.

This species was threatened by the marine curio trade and listed as a protected species in Sri Lanka under the Sri Lankan Fauna and Flora Protection Act (Amendment), No. 22 of 2009. To date, it is the only protected echinoid species in Sri Lanka.

**Family Temnopleuridae** A. Agassiz, 1872

***Microcyphus ceylanicus*** Mortensen, 1942

Figure 2

**Material studied.** WUSL/ER/87 (dry, denuded) from Hiriketiya; WUSL/ER/234 (wet, with spines) from Hiriketiya; WUSL/ER/235 (dry, denuded) from Dickwella.

**Literature records for Sri Lanka.** Mortensen (1942, 1943a), Price & Rowe (1996), Fernando (2006), Jayakody (2012).

**Distribution in Sri Lanka.** Southern coast of Sri Lanka.

**Recorded depth range in Sri Lanka.** 1–5 m (previous records).

**Habitat.** Coral reefs (Price & Rowe 1996).

**Observed occurrence in this study.** Southern coast (Hiriketiya and Dickwella), on shore. Both of these collection sites were dominated by seagrass beds at shallow depths.

**Figure 2:**
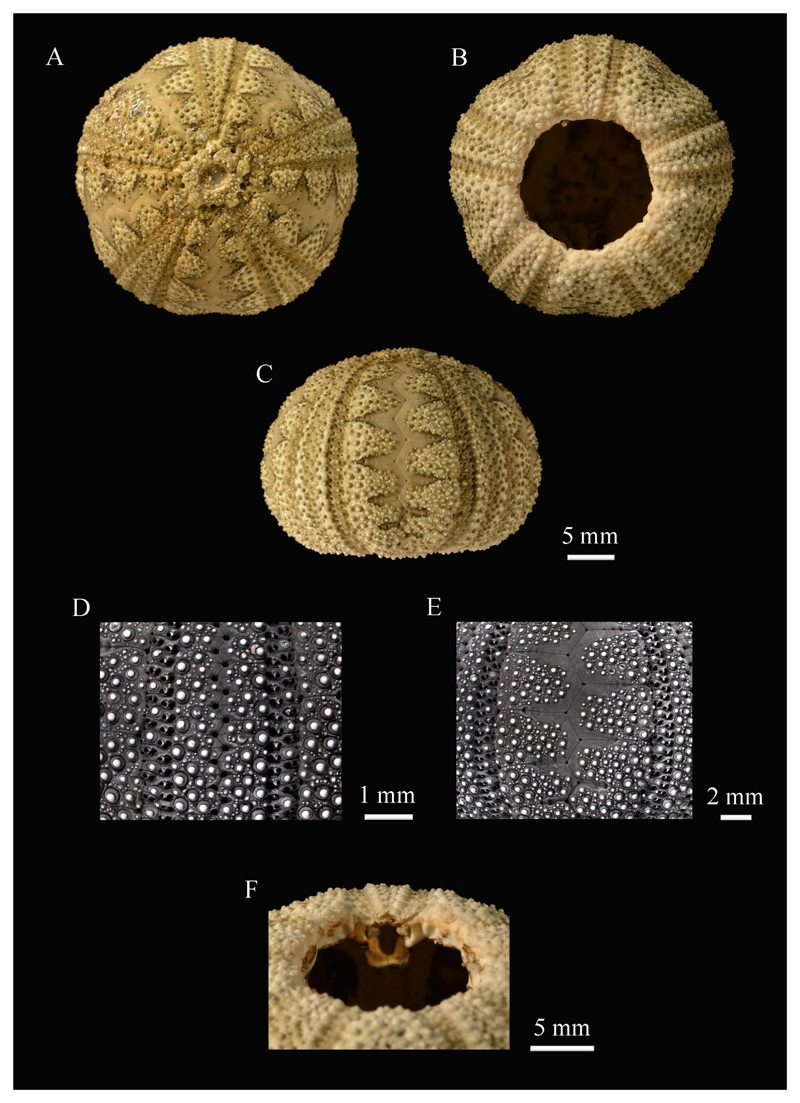
Test features of *Microcyphus ceylanicus* (WUSL/ER/87): A, aboral view; B, oral view; C, lateral view; D, ambital ambulacrum; E, ambital interambulacrum; F, oblique view through peristome showing an auricle D and E whitened with ammonium chloride.

**Remarks.** Here, for the first time, we provide photographs of test surface details on this rarely seen temnopleurid (Fig. 2). Its test has never been figured in detail, and Mortensen (1943a) included only a photograph in lateral view, plus drawings of the apical system, ambulacral compounding, and some pedicellariae. *M. ceylanicus* is restricted to Sri Lanka and the Andaman Islands, and can be distinguished from other Sri Lankan regular echinoids by its light olive-green test. The test has naked interambulacral areas, each of which has a dark zigzag line along the medial sutures (Fig. 2). The spines are banded with red, brown, and white. This species is characterized by its sharply delimited naked areas in both the interambulacra and ambulacra (Mortensen 1942).

Döderlein (1888) misidentified this species as *M. maculatus*, an error that was rectified by Mortensen (1943a). The type specimen, collected from Sri Lanka, is housed at the Zoologische Staatssammlung München (The Bavarian State Collection of Zoology, Munich).

***Salmacis bicolor* L. Agassiz in** L. Agassiz & Desor, 1846

Figure 3

**Material studied.** WUSL/ER/236, 237, 238 (wet, with spines) from Godawaya; WUSL/ER/239 (dry, with spines) from Nilwella; WUSL/ER/240 (wet, with spines) and WUSL/ER/241 (dry, with spines) from Mandathiv; WUSL/ER/242 (wet, with spines) and WUSL/ER/88, 243 (dry, with spines) from Nagadeepaya; WUSL/ER/244, 245 (dry, with spines) from Point Pedro.

**Literature records for Sri Lanka.** Bell (1882, 1887), Döderlein (1888), Herdman *et al.* (1904), Clark (1915, 1925), Koehler (1927), Price & Rowe (1996), Schultz (2005), Fernando (2006), Sastry (2007), Jayakody (2012).

**Distribution in Sri Lanka.** Northern, southern, and western coasts of Sri Lanka.

**Recorded depth range in Sri Lanka.** 1–5 m (present study), 4–55 m (previous records).

**Habitat.** Rocky shores, among boulders and seagrass.

**Observed occurrence in this study.** Northern coast (Mandathiv, Nagadeepa, and Point Pedro) and the southern coast (Godawaya and Nilwella) of Sri Lanka.

**Figure 3:**
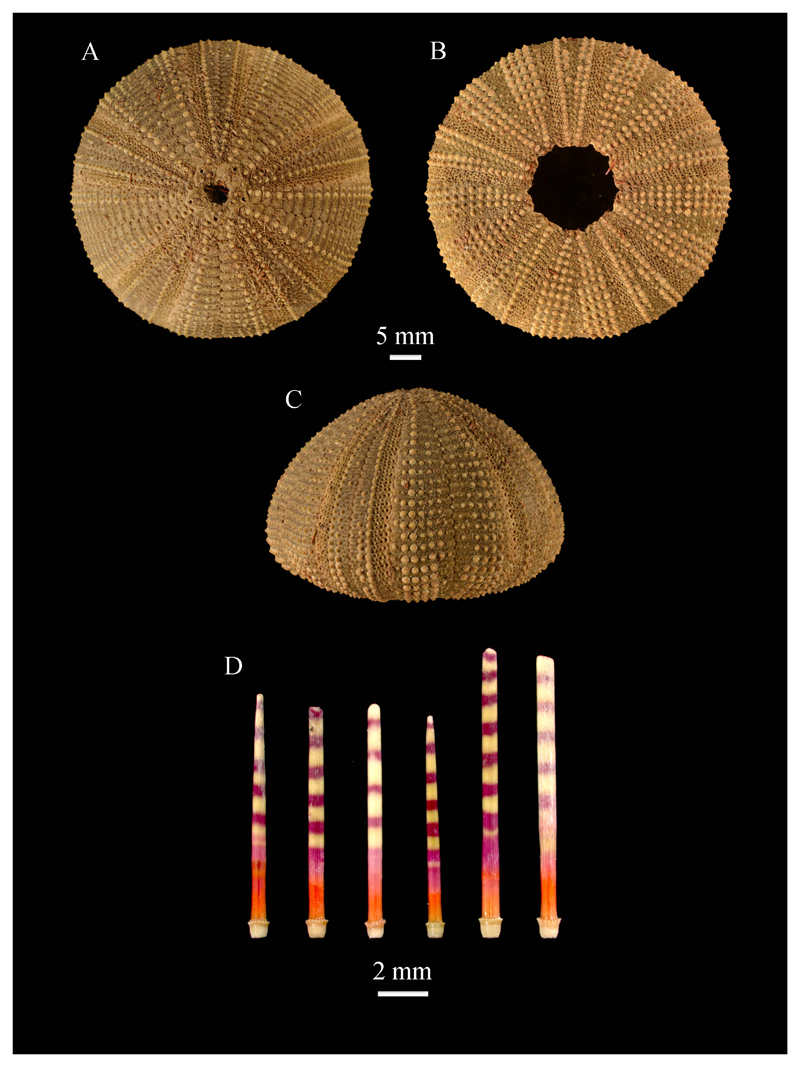
Test features and spines of *Salmacis bicolor* (WUSL/ER/88): A, aboral view; B, oral view; C, lateral view; D, ambital spines.

**Remarks.** *S. bicolor* differs from other species in the genus in that the spines are banded in red and yellowish to violet or green and have red bases (Fig. 3). This species is well documented in Sri Lanka.

***Salmacis virgulata* L. Agassiz in** L. Agassiz & Desor, 1846

Figure 4

**Material studied.** WUSL/ER/246, 247 (wet, with spines) and WUSL/ER/89, 248, 249, 250 (dry, with spines) from Nagadeepaya; WUSL/ER/251 (wet, with spines) and WUSL/ER/252 (dry, with spines) from Mandathiv; WUSL/ER/253, 254 (dry, with spines) from Mulathiv; WUSL/ER/255, 256 (dry, with spines) from Point Pedro; WUSL/ER/257, 258 (dry, with spines) from Silavathurai.

**Literature records for Sri Lanka.** Agassiz & Desor (1846), Clark (1915, 1925), Koehler (1927), Fernando (2006), Jayakody (2012).

**Distribution in Sri Lanka.** Northern, southern, and northwestern coasts of Sri Lanka.

**Recorded depth range in Sri Lanka.** 9–12 m (present study), 59 m (previous records).

**Habitat.** Among seagrass beds and coral rubble.

**Observed occurrence in this study.** Northern coast (Mandathiv, Mulathiv, Nagadeepa, Point Pedro, and Silavathurai) of Sri Lanka.

**Figure 4:**
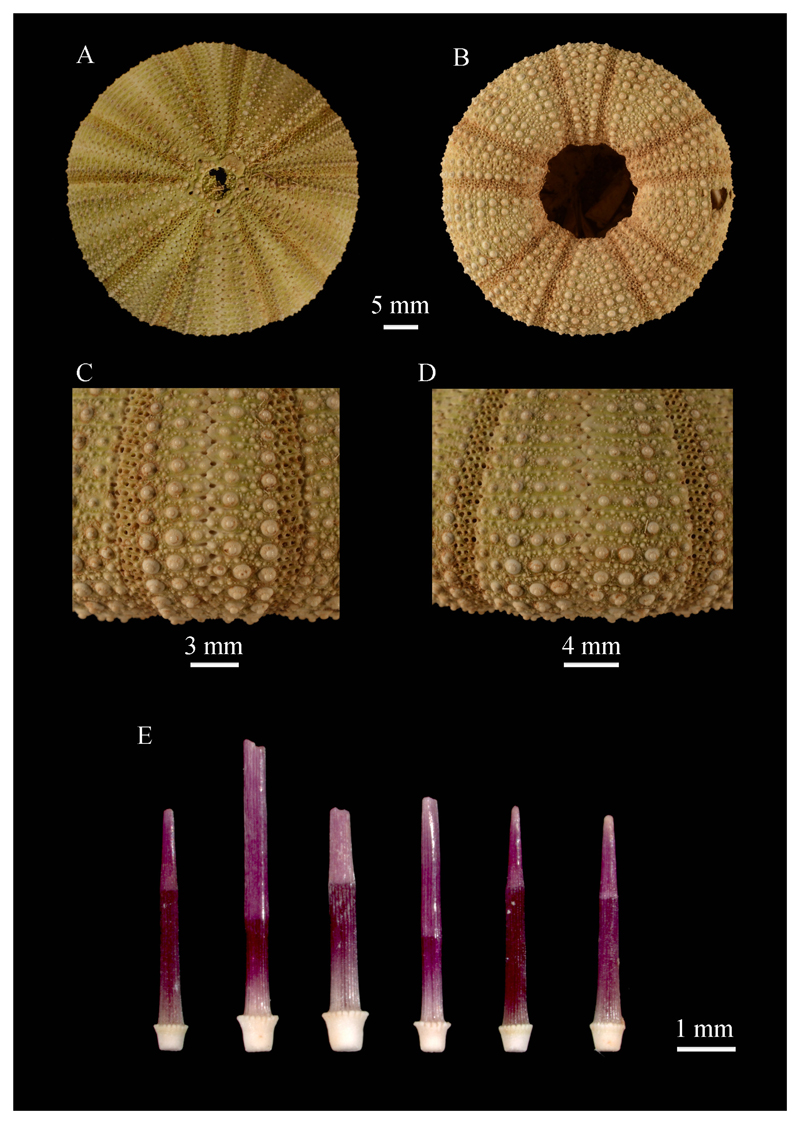
Test features and spines of *Salmacis virgulata* (WUSL/ER/89): A, aboral view; B, oral view; C, ambital ambulacrum; D, ambital interambulacrum; E, ambital spines.

**Remarks.** *S. virgulata* can be distinguished from others in the genus in having uniformly purplish, unbanded primary spines with whitish bases (Fig. 4).

This was the first echinoid species recorded to occur in Sri Lanka by Agassiz & Desor (1846), who cited the locality as “Ceylan” (Sri Lanka) for “*Salmacis virgulatus*”, an incorrect formulation of the name. The holotype (EcEh 5940) is from Sri Lanka, and is housed at the Muséum National d’Histoire Naturelle, France (Vadon *et al.* 1984).

***Temnopleurus toreumaticus* (Leske, 1778)**

Figure 5

**Material studied.** WUSL/ER/259, 260, 261 (wet, with spines) and WUSL/ER/95, 262, 263, 264 (dry, with spines) from Nagadeepaya; WUSL/ER/265, 266 (wet, with spines) and WUSL/ER/267 (dry, with spines) from Mandathiv; WUSL/ER/268 (wet, with spines) and WUSL/ER/269, 270 (dry, with spines) from Point Pedro.

**Literature records for Sri Lanka.** Agassiz & Desor (1846), Agassiz (1872), Bell (1887), Anderson (1894), Herdman *et al.* (1904), Clark (1915, 1925), Koehler (1927).

**Distribution in Sri Lanka.** Northern and southern coasts of Sri Lanka.

**Recorded depth range in Sri Lanka.** 9–66 m (previous records).

**Habitat.** Bottoms consisting of “*Orbitolites* sand, some dead coral, shells and pieces of *Nullipore*” [sic] (Herdman *et al.* 1904).

**Observed occurrence in this study.** Fish landing sites at Mandathiv, Nagadeepa, and Point Pedro on the northern coast of Sri Lanka.

**Figure 5:**
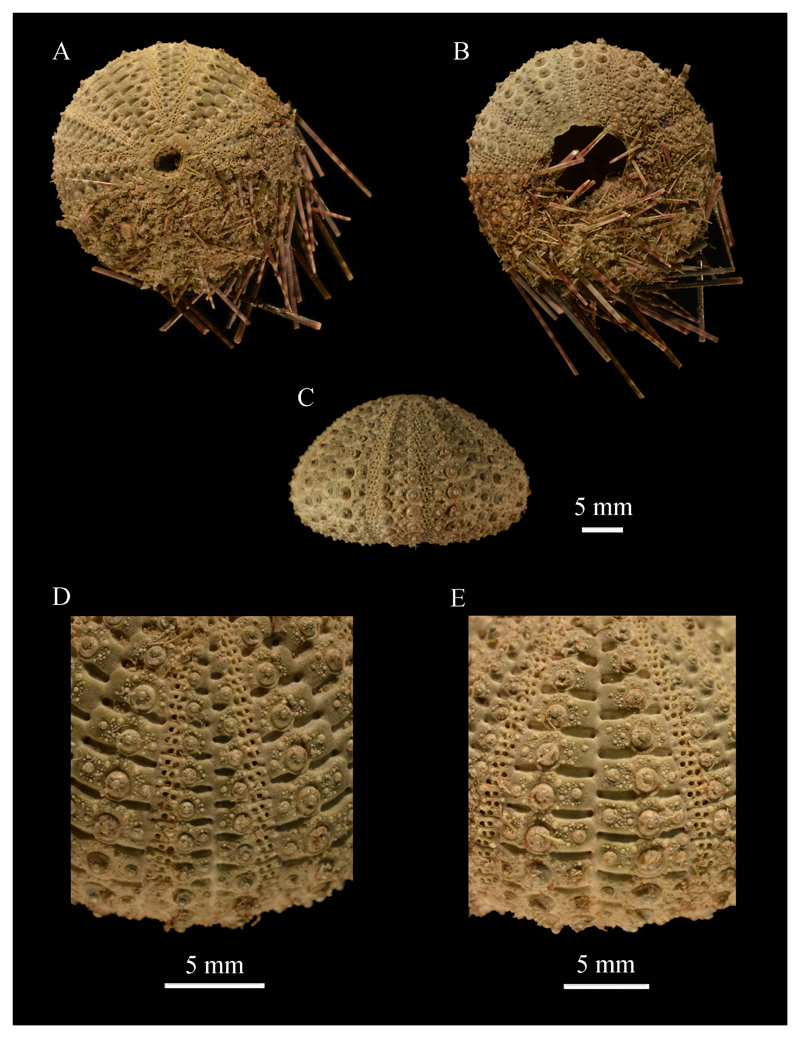
Test features and spines of *Temnopleurus toreumaticus* (WUSL/ER/95): A, aboral view (with spines); B, oral view (with spines); C, lateral view; D, adapical ambulacrum; E, adapical interambulacrum.

**Remarks.** This species can be distinguished from other Sri Lankan temnopleurids in having conspicuous, deep, long furrows along the plate sutures (so-called sutural pits). These furrows extend horizontally to the bases of the primary tubercles (Fig. 5).

**Family Toxopneustidae Troschel, 1872**

***Pseudoboletia maculata* Troschel, 1869**

**Material studied.** WUSL/ER/271 (dry, with spines) from Polhena.

**Literature records for Sri Lanka.** Herdman *et al.* (1904), Clark (1915), Fernando (2006), Jayakody (2012).

**Distribution in Sri Lanka.** Northern and southern coasts of Sri Lanka.

**Recorded depth range in Sri Lanka.** 13 m (previous records).

**Habitat.** Not recorded.

**Observed occurrence in this study.** None.

**Remarks.** *P. maculata* differs from *P. indiana* because the former has dark spots on the test, but the latter is uniform in colour and lacks spots.

One denuded specimen is housed at the DAF (Polhena, 1–5 m).

***Toxopneustes pileolus* (Lamarck, 1816)**

**Material studied.** WUSL/ER/273 (wet, with spines) from Silavathurai and WUSL/ER/274 (dry, with spines) from Polhena; WUSL/ER/275 (dry, denuded) from Negombo.

**Literature records for Sri Lanka.** Walter (1885), Döderlein (1888), Herdman *et al.* (1904), Clark (1915), Koehler (1927), Fernando (2006), Jayakody (2012).

**Distribution in Sri Lanka.** Northern, southern, and western coasts of Sri Lanka.

**Recorded depth range in Sri Lanka.** 0.5–12 m (present study), 13–48 m (previous records).

**Habitat.** Rocky reef areas, seagrass beds.

**Observed occurrence in this study.** Northern (Silavathurai), southern (Polhena, Hikkaduwa, Hiriketiya, Ahangama), and western coasts (Negombo) of Sri Lanka.

**Remarks.** When the animal is alive, the test takes on the appearance of a flower garden because of the dense covering of large, bright reddish and white, three-jawed globiferous pedicellariae. In contrast, the denuded test has distinct greenish to purplish bands arranged concentrically. This species can be distinguished from the other two toxopneustid species recorded in this study, *Pseudoboletia maculata* and *Tripneustes gratilla gratilla*, because the former has clear arcs of three pore pairs each in the ambulacra, and the latter has denser tuberculation and lacks the green banding on the denuded test (in addition, its test is much higher and shows three discrete vertical series of pore pairs in each ambulacral column rather than a broad band of pores).

***Tripneustes gratilla gratilla* (Linnaeus, 1758)**

**Material studied.** WUSL/ER/276 (wet, denuded) from Silavathurai; WUSL/ER/277 (wet, with spines) and WUSL/ER/278 (wet, denuded) from Hiriketiya; WUSL/ER/279 (dry, denuded) from Polhena; WUSL/ER/280 (dry, denuded) from Hikkaduwa; WUSL/ER/281 (dry, denuded) from Ahangama.

**Literature records for Sri Lanka.** Walter (1885), Döderlein (1888), Clark (1915), Koehler (1927), Fernando (2006), Jayakody (2012).

**Distribution in Sri Lanka.** Southern, northern, and northwestern coasts of Sri Lanka.

**Recorded depth range in Sri Lanka.** 0.1–12 m (present study).

**Habitat.** Mainly found on sandy bottoms among seagrass beds.

**Observed occurrence in this study.** Northern (Silavathurai), southern (Polhena, Hikkaduwa, Hiriketiya, Kirinda, Godawaya, Ahangama), and western coasts (Negombo) of Sri Lanka.

**Remarks.** *Tripneustes gratilla gratilla* is one of the most common shallow water species found among the seagrass beds of the southern coast of Sri Lanka. The colour of the primary spines varies from orange to white.

## Discussion

This updated checklist records 39 regular echinoid taxa found in the waters around Sri Lanka. The present research added no new records or new species to the most recent checklist compiled by Arachchige *et al.* (2017). However, it does provide confirmation for occurrence in Sri Lanka of *Phyllacanthus imperialis* and *Temnopleurus toreumaticus* since their last recorded sightings 90 years ago (see Arachchige *et al.* 2017). Out of the 39 echinoid taxa, 16 species had been recorded in the literature from waters deeper than 30 m and as such, were considered deeper water regular echinoid species (Table 1). *Stylocidaris albidens* and *Desmechinus versicolor* were also included with the deep-water regulars using the criteria of Schultz (2011), although precise depths were not recorded. Out of the 16 deep-water species recorded in the previous studies, only one species, *Salmacis virgulata*, was recorded during this study because of our focus on shallow-water species. Distribution data for *Acanthocidaris* sp., *Stylocidaris albidens*, *Colobocentrotus* (*Podophora*) *atratus* and *Salmacis belli* could not be found during the fieldwork and these species were historically mentioned solely with the indication “Ceylon or Sri Lanka” in the literature.

Based on the records of Clark (1925), *Colobocentrotus* (*Podophora*) *atratus* was included in the present species list. However, Clark does not cite an exact location, collector, or any other description, providing only a citation in a list of species found in the collection of the Museum of Natural History, UK and giving the distribution of *C.* (*P.*) *atratus* as “Ceylon”. As this is a littoral species restricted to the surf zone (Mortensen 1943a), it is not clear how a commonly reported littoral species has remained entirely unknown to other collectors in Sri Lanka apart from Clark’s single record. This situation could pertain if the species is very rare, even though it is littoral in Sri Lankan waters. Therefore, there remains no verified presence of this species in Sri Lanka and the reported locality “Ceylon” may be attributed to an erroneous label. *C.* (*P.*) *atratus* is well known from Kenya in the western Indian Ocean (Samyn 2003) and the Andaman Sea in the eastern Indian Ocean (Putchakarn & Sonchaeng 2004; Sastry 2007), suggesting that it could potentially occur in Sri Lanka. However, it has not been recorded from the southeastern Arabian Sea (Parameswaran *et al.* 2017).

In addition to the above species, *Phyllacanthus forcipulatus*, *Prionocidaris bispinosa*, *Stereocidaris indica*, *Sperosoma biseriatum*, *Phormosoma bursarium*, *Salmacis belli*, *S. roseoviridis*, *Temnotrema siamense*, *Pseudoboletia indiana*, and *Desmechinus versicolor* have been collected only once from Sri Lankan waters.

*P. forcipulatus* is also known in the Indian Ocean from Madras, India (Schultz 2011). On the other hand, *S. indica* is well known throughout the Indo-Pacific from the Arabian Sea to the Philippines and Japan (Mortensen 1928; Schultz 2011), as well as the Andaman Sea (Sastry 2007). Similarly, *S. biseriatum* has been recorded from the west of Sri Lanka in the Laccadive Sea by Koehler (1927). This species is also known from Kenya, the Arabian Sea, and South Africa (Clark & Courtman-Stock 1976). *P. bursarium* is well known from the southeastern Arabian Sea (Parameswaran *et al.* 2017) and the Andaman Sea (Sastry 2007). *P. indiana* is recorded from the Indian Ocean from eastern Africa and Madagascar (Clark & Rowe 1971). *T. siamense* is well known from the Indian Ocean from the Arabian Sea (Samyn 2003) to the Andaman Sea (Putchakarn & Sonchaeng 2004). Conversely, *S. roseoviridis* is only known from the Indian Ocean from off Sri Lanka and off the coast of Myanmar (Burma) (Mortensen 1943a; Schultz 2011). The known ranges of these species suggest that there is a high probability of occurrence of these species in Sri Lankan waters, thus making it unlikely that the single records of the species discussed in this paragraph are all based on misidentifications.

*P. bispinosa, S. belli*, and *D. versicolor* have been recorded only once from Sri Lankan waters. These are the only records available for the entire Indian Ocean.

Herdman *et al.* (1904) recorded *P. bispinosa* from Sri Lankan waters. This is the only available distribution record in Clark and Rowe (1971) for this species in the Indian Ocean. However, this species is known from the Gulf of Thailand (Putchakarn & Sonchaeng 2004). *S. belli* has been documented from Sri Lankan waters only by Jayakody (2012). This species is distributed widely in the Pacific Ocean from the Philippines, the Malayan Archipelago, and the northern coast of Australia (Clark & Rowe 1971; Miskelly 2002; Mooi & Munguia 2014; Schultz 2005). The only available record for *D. versicolor* in the Indian Ocean was given by Mortensen (1943a: 345, 346). He provided coordinates for a single specimen collected from off south Sri Lanka by the R.I.M.S. “*Investigator*”. This species is known from the Indo-Pacific, specifically from the Kei Islands, Indonesia to the China Sea (Mortensen 1943a). The ranges of all these species are consistent with the possibility that future surveys will confirm the presence of *P. bispinosa*, *S. belli*, and *D. versicolor* in Sri Lanka.

No management plan for the conservation and sustainable utilization of any taxon can be implemented successfully without basic biological and ecological information. Because echinoids are rapidly becoming exploited commercially as a marine delicacy (Scheibling & Mladenov 1987; Johnson *et al.* 2012) and are exported as decorative objects, it is time to evaluate the current status of sea urchins in Sri Lanka, and to develop new identification guides for the use of stakeholders in these growing industries. Reliable taxonomic data are also required to fill gaps in our knowledge of the ecological roles of echinoids along Sri Lankan shores. The data in the present study can be used for future work on the regular echinoid fauna of Sri Lanka, particularly in the assessment of population sizes, spatial distribution, local trophic networks, and threats to biodiversity due to natural and anthropogenic changes. Furthermore, systematic deep-water surveys are needed to increase our knowledge of echinoid species diversity in Sri Lankan waters.

## Supplementary Material

Appendices

## Figures and Tables

**Table 1:** Annotated checklist of regular echinoids of Sri Lanka. Explanation of symbols: bold font indicates species recorded during the fieldwork within the present study or available in the DAF collection; L—indicates species recorded in literature; P—indicates species recorded in the present study; E—eastern coast; GM—Gulf of Mannar; N—northern coast; NW—northwestern coast; S—southern coast; W—western coast; 0 (zero) in depth column indicates specimens found at the beach or at fish landing site; I—Sarasin & Sarasin (1886, 1887, 1888), Sarasin (1888); II—de Loriol (1874); III—Anderson (1894); IV—Herdman *et al.* (1904); V—Clark (1925); VI—Koehler (1927); VII—Mortensen (1943a); VIII—Price & Rowe (1996); IX—Sastry (2007); X—Gayashan & Jayakody (2012); XI—California Academy of Sciences.

Taxa	Distribution data from literature*	Distribution data from this study	Depth (m) records in literature	Depth (m) records in this study	Source for literature distribution data
Order Cidaroida Claus, 1880					
Family Cidaridae Gray, 1825					
*Acanthocidaris* sp.					
*Eucidaris metularia* (Lamarck, 1816)	GM, S		62–65		IV, VI
*Phyllacanthus forcipulatus* Mortensen, 1936	S		95–124		IX
***Phyllacanthus imperialis* (Lamarck, 1816)**	GM	S, W	23–65	1–8	IV
*Prionocidaris baculosa* (Lamarck, 1816) [also recorded as *P. baculosa annulifera* (A. Agassiz, 1873)]	GM, N, S, W		7–182		II, IV, VI
*Prionocidaris bispinosa* (Lamarck, 1816)	GM				IV
*Stereocidaris indica* Döderlein, 1901	W		733		VI
*Stylocidaris albidens* H.L. Clark, 1925					V
*Stylocidaris tiara* (Anderson, 1894)	W		329–397		III, VI
Order Echinothurioida Claus, 1880					
Family Echinothuriidae Thomson, 1872					V
*Araeosoma coriaceum indicum* Koehler, 1921	W		733		VI
*Asthenosoma varium* Grube, 1868	E				I, V
*Sperosoma biseriatum* Döderlein, 1901	W		836–1077		VI
Family Phormosomatidae Mortensen, 1934					
*Phormosoma bursarium* A. Agassiz, 1881	W		733		VI
Order Diadematoida Duncan, 1889					
Family Diadematidae Gray, 1855					
***Astropyga radiata* (Leske, 1778)**	E		51		I, III
***Diadema savignyi* (Audouin, 1809)**	S	S		0.5–4	X
***Diadema setosum* (Leske, 1778)**	S, E	S	0–62	0.5–3	I, IV, VIII, X
***Echinothrix calamaris* (Pallas, 1774)**		S		1–2	
***Echinothrix diadema* (Linnaeus, 1758)**	S	S	62	1–2	IV
Order Stomopneustoida Kroh & Smith 2010					
Family Stomopneustidae Mortensen, 1903					
***Stomopneustes variolaris* (Lamarck, 1816)**	S	E, NW, S, W	5	0.1–5	VIII, IV, X, XI
Order Camarodonta Jackson, 1912					
Family Echinometridae Gray, 1855					
*Colobocentrotus* (*Podophora*) *atratus* (Linnaeus, 1758)					V
***Echinometra* ex. grupo *mathaei* (Blainville, 1825)**	NW, S	S, W	0.5–5	0.5–1	VIII, X
*Echinometra oblonga* (Blainville, 1825)	S		1–5		XI
***Echinostrephus molaris* (Blainville, 1825)**	GM, S	NW, S	13–24	0.5–13	IV, X
***Heterocentrotus mamillatus* (Linnaeus, 1758)**		S		2–5	
Family Temnopleuridae A. Agassiz, 1872					
***Microcyphus ceylanicus*** Mortensen, 1942	NW	S	1–5	0	VIII
*Salmaciella dussumieri* (L. Agassiz in L. Agassiz & Desor, 1846)	GM, S, W		13–66		IV, VI, IX
*Salmacis belli* Döderlein, 1902					Jayakody (2012)
***Salmacis bicolor* L. Agassiz in** L. Agassiz & Desor, 1846	GM, S, W	N, S	4–55	0, 1–5	IV, VIII, VI
*Salmacis roseoviridis* Koehler, 1927	W				VI
***Salmacis virgulata* L. Agassiz in** L. Agassiz & Desor, 1846	S	N	59	0, 9–12	VI
*Temnopleurus* sp.	GM, S, W		13–22		IV
***Temnopleurus toreumaticus*** (Leske, 1778)	GM, N, S	N	9–66	0	III, IV, VI
*Temnotrema siamense* (Mortensen, 1904)	S		62		VI
Family Toxopneustidae Troschel, 1872					
*Gymnechinus robillardi* (de Loriol, 1883)	S		48–59		VI
*Pseudoboletia Indiana* (Michelin, 1862)	S		62		VI
***Pseudoboletia maculata* Troschel, 1869**	GM		13		IV
***Toxopneustes pileolus* (Lamarck, 1816)**	GM	N, S	13–48	0.5–12	IV
***Tripneustes gratilla gratilla* (Linnaeus, 1758)**	S	N, S		0.1–12	X
Family Trigonocidaridae Mortensen, 1903					
*Desmechinus versicolor* (Mortensen, 1904)	S				VII
Total Taxa 39					

* See Appendix 2 for explanation.

## References

[R1] Agassiz A (1872–1874). Revision of the Echini. Illustrated Catalogue of the Museum of Comparative Zoölogy at
Harvard College.

[R2] Agassiz L, Desor PJE (1846). Catalogue raisonné des familles, des genres, et des
espèces de la classe des échinodermes. Annales des Sciences Naturelles, Troisième Série,
Zoologie.

[R3] Anderson ARS (1894). Natural history notes from the H. M. Indian Marine Survey Steamer
“Investigator”, Commander C. F. Oldham, R. N., commanding.
Series II, No. 16. On the Echinoidea collected during the season
1893–94. Journal of the Asiatic Society of Bengal.

[R4] Appeltans W, Ahyong ST, Anderson G, Angel MV, Artois T, Bailly N, Bamber R, Barber A, Bartsch I, Berta A, Blazewicz-Paszkowycz M (2012). The magnitude of global marine species diversity. Current Biology.

[R5] Arachchige GM, Jayakody S, Mooi R, Kroh A (2017). A review of previous studies on the Sri Lankan echinoid fauna,
with an updated species list. Zootaxa.

[R6] Arachchige GM, Jayakody S, Mooi R, Kroh A (2019). Taxonomy and distribution of irregular echinoids (Echinoidea:
Irregularia) of Sri Lanka. Zootaxa.

[R7] Barnes DKA, Verling E, Crook A, Davidson I, O’Mahoney M (2002). Local population disappearance follows (20 yr after) cycle
collapse in a pivotal ecological species. Marine Ecology Progress Series.

[R8] Bell FJ (1882). Note on the echinoderm-fauna of the island of Ceylon, together
with some observations on heteractinism, XIX. Annals and Magazine of Natural History, Series 5.

[R9] Bell FJ (1887). The echinoderm fauna of the island of Ceylon. Scientific Transactions of the Royal Dublin Society.

[R10] Bronstein O, Georgopoulou E, Kroh A (2017). On the distribution of the invasive long-spined echinoid Diadema
setosum and its expansion in the Mediterranean Sea. Marine Ecology Progress Series.

[R11] Cebrian E, Uriz MJ (2006). Grazing on fleshy seaweeds by sea urchins facilitates sponge
*Cliona viridis* growth. Marine Ecology Progress Series.

[R12] Clark AM, Courtman-Stock J (1976). The Echinoderms of Southern Africa. Publication No. 766.

[R13] Clark AMG, Rowe FWE (1971). Monograph of Shallow-water Indo-West Pacific Echinoderms.

[R14] Clark HL (1907). The Cidaridae. Bulletin of the Museum of Comparative Zoology at Harvard
College.

[R15] Clark HL (1915). The echinoderms of Ceylon other than holothurians. Spolia Zeylanica.

[R16] Clark HL (1917). Hawaiian and other Pacific Echini, Echinoneidae, Nucleolitidae,
Urechinidae, Echinocorythidae, Calymnidae, Pourtalesiidae, Palaestomatidae,
Aeropsidae, Palaeopneustidae, Hemiasteridae, and Spatangidae. Memoirs of the Museum of Comparative Zoology at Harvard College.

[R17] Clark HL (1925). A Catalogue of the Recent Sea-Urchins (Echinoidea) in the Collection of
the British Museum (Natural History).

[R18] Coppard SE, Campbell AC (2006). Taxonomic significance of test morphology in the echinoid genera
*Diadema* Gray, 1825 and *Echinothrix*
Peters, 1853 (Echinodermata). Zoosystema.

[R19] David B, Mooi R, Néraudeau D, Saucède T, Villier L (2009). Évolution et radiations Adaptatives adaptatives chez les
échinides. Comptes Rendus-Palevol.

[R20] Döderlein L (1888). Echinodermen von Ceylon. Bericht über die von den Herren
D^res^ Sarasin gesammelten Asteroidea, Ophiuroidea und
Echinoidea. Zoologische Jahrbücher, Abteilung für Systematik,
Geographie und Biologie der Tiere.

[R21] Durham JW, Wagner CD, Moore RC (1966). Glossary of morphological terms applied to
echinoids. Treatise on Invertebrate Paleontology. Part U. Echinodermata 3.
Echinozoa, Echinoidea.

[R22] Fauna and Flora Protection (2009). Fauna and Flora Protection (Amendment) Act, No. 2 of
2009. Gazette of the Democratic Socialist Republic of Sri Lanka of
April.

[R23] Fernando M, Bambaradeniya CNB (2006). Coral associated invertebrates: An overview of the current
taxonomic status. The Fauna of Sri Lanka. Section 3. Status of Marine Fauna in Sri
Lanka.

[R24] Gayashan MA, Jayakody S (2012). Diversity and density of sea urchins populations in rocky shores
off Nilwella in Southern province of Sri Lanka. Sri Lanka Journal of Aquatic Science.

[R25] Hardy C, David B, Rigaud T, De Ridder C, Saucède T (2011). Ectosymbiosis associated with cidaroids (Echinodermata:
Echinoidea) promotes benthic colonization of the seafloor in the Larsen
Embayments, Western Antarctica. Deep Sea Research II.

[R26] Herdman WA, Herdman JB, Bell FJ, Herdman WA (1904). Report on the Echinoderma collected by Professor Herdman, at
Ceylon, in 1902. Report to the government of Ceylon on the pearl oyster fisheries of the
Gulf of Mannar.

[R27] Jayakody S, Weerakoon DK, Wijesundara S (2012). Provisional checklist of sea urchins (Echinodermata: Echinoidea)
of Sri Lanka. The National Red List 2012 of Sri Lanka; Conservation Status of the
Fauna and Flora.

[R28] Johnson TR, Wilson JA, Cleaver C, Vadas RL (2012). Social-ecological scale mismatches and the collapse of the sea
urchin fishery in Maine, USA. Ecology and Society.

[R29] Koehler R (1927). Echinoderma of the Indian Museum 10. An account of the Echinoidea. 3.
Echinides réguliers.

[R30] Kroh A, Mooi R (2018). World Echinoidea Database. http://www.marinespecies.org/echinoidea/.

[R31] Kroh A, Smith AB (2010). The phylogeny and classification of post-Palaeozoic
echinoids. Journal of Systematic Palaeontology.

[R32] Lessios HA, Kessing BD, Pearse JS (2001). Population structure and speciation in tropical seas: global
phylogeography of the sea urchin *Diadema*. Evolution.

[R33] de Loriol P (1874). Description de trois espèces d’Échinides
appartenant à la famille des Cidaridées. Mémoires de la Société des Sciences Naturelles de
Neuchâtel.

[R34] Melville R, Durham JW, Moore RC (1966). Skeletal morphology. Treatise on Invertebrate Paleontology. Part U. Echinodermata 3.
Echinozoa, Echinoidea.

[R35] Miskelly A (2002). Sea urchins of Australia and the Indo-Pacific.

[R36] Mooi R, Munguia A, Williams GC, Terrence MG (2014). Sea urchins of the Philippines. The Coral Triangle: The 2011 Hearst Philippine Biodiversity
Expedition.

[R37] Mortensen T (1928). A Monograph of the Echinoidea. I. Cidaroidea.

[R38] Mortensen T (1935). A Monograph of the Echinoidea. II. Bothriocidaroida, Melonechinoida,
Lepidocentroida and Stirodonta.

[R39] Mortensen T (1940). A Monograph of the Echinoidea. III. 1. Aulodonta. Lepidocentroida and
Stirodonta.

[R40] Mortensen T (1942). New Echinoidea (Camarodonta). Preliminary notice. Videnskabelige Meddelelser fra Dansk naturhistorisk Forening i
København.

[R41] Mortensen T (1943a). A Monograph of the Echinoidea III. 2. Camarodonta I. Orthopsidæ,
Glyphocyphidæ, Temnopleuridæ and Toxopneustidæ.

[R42] Mortensen T (1943b). A Monograph of the Echinoidea III.3, Camarodonta. II. Echinidæ,
Strongylocentrotidæ, Parasaleniidæ,
Echinometridæ.

[R43] Parameswaran UV, Sanjeevan VN, Jaleel KA, Jacob V, Gopal A, Vijayan AK, Sudhakar M (2017). An updated checklist of echinoderms of the southeastern Arabian
Sea. Marine Biodiversity.

[R44] Pawson DL (2007). Phylum Echinodermata. Zootaxa.

[R45] Price ARG, Rowe FW (1996). Indian Ocean echinoderms collected during the Sindbad Voyage
(1980–81), 3. Ophiuroidea and Echinoidea. Bulletin of the Natural History Museum, Zoology Series.

[R46] Putchakarn S, Sonchaeng P (2004). Echinoderm fauna of Thailand: History and inventory
reviews. Science Asia.

[R47] Samyn Y (2003). Shallow-water regular echinoids (Echinodermata: Echinoidea) from
Kenya. African Zoology.

[R48] Sarasin CF, Sarasin PB (1886). Über einen Lederigel aus dem Hafen von Trincomalie
(Ceylon) und seinen Giftapparat. Zoologischer Anzeiger.

[R49] Sarasin F (1888). Ueber *Asthenosoma urens*, einen Echinothuriden
von Trincomali. Sitzungsberichte der Gesellschaft Naturforschender Freunde zu
Berlin.

[R50] Sarasin P, Sarasin F (1887). Die Augen und das Integument der Diadematiden. Ergebnisse Naturwissenschaftlicher Forschungen auf Ceylon.

[R51] Sarasin P, Sarasin F (1888). Ueber die Anatomie der Echinothurien und die Phylogenie der
Echinodermen. Ergebnisse Naturwissenschaftlicher Forschungen auf Ceylon.

[R52] Sastry DRK (2007). Echinodermata of India, an annotated list. Records of the Zoological Survey of India. Occasional Paper.

[R53] Saucède T, Mooi R, David B (2007). Phylogeny and origin of Jurassic irregular echinoids
(Echinodermata: Echinoidea). Geological Magazine.

[R54] Scheibling RE, Mladenov PV (1987). The decline of the sea urchin, *Tripneustes
ventricosus*, fishery of Barbados: A survey of fishermen and
consumers. Marine Fisheries Review.

[R55] Schultz H (2005). Sea Urchins: A Guide to Worldwide Shallow Water Species.

[R56] Schultz H (2011). Sea-Urchins lll, Worldwide Regular Deep Water Species.

[R57] Serafy DK, Fell FJ (1985). Marine flora and fauna of the northeastern United States:
Echinodermata: Echinoidea. National Oceanic and Atmospheric Administration Technical Reports,
National Marine Fisheries Service.

[R58] Smith AB, Kroh A (2011). The Echinoid Directory. World Wide Web electronic publication.

[R59] Vadon C, de Ridder C, Guille A, Jangoux M (1984). Les types d’Échinides actuels (Échinodermes)
du Múseum national d’Histoire naturelle de
Paris. Bulletin du Muséum national d’Histoire naturelle, 4e
série, Section A (Zoologie, Biologie et Écologie
animales).

[R60] Walter A (1885). Ceylons Echinodermen. Jenaische Zeitschrift für Naturwissenschaft Herausgegeben von der
Medicinisch-Naturwissenschaftlichen Gesellschaft zu Jena.

