# Supplementary material for: An annotated species list of regular echinoids from Sri Lanka with notes on some rarely seen temnopleurids
Source: Zootaxa. Author manuscript; Available in PMC 2019 Dec 13. (PMC6910863; doi:10.11646/zootaxa.4571.1.3)
Supplement: Appendices [file EMS85012-supplement-Appendices.pdf]

APPENDIX 1. Sampling sites for this study. Explanation of symbols: E—east coast; N—north coast; NW—northwestern coast; S—south coast; W—west coast; 0 (zero) in depth column indicates specimens found on shore or at fish landing site.

Site Code	Location	Site name	Province	Latitude	Longitude	Depth range (m)
E	Eastern coast of Sri Lanka	Batticaloa	Eastern	7°43′08″N	81°43′31″E	1–5
		Trincomalee	Eastern	8°33′54″N	81°14′29″E	3–4
		Panama	Eastern	6°46′05″N	81°49′30″E	1–3
N	Northern coast of Sri Lanka	Mandathiv	Northern	9°35′54″N	79°58′50″E	0
		Mannar	Northern	8°53′37″N	79°55′43″E	0
		Mulathiv	Northern	9°22′41″N	80°42′42″E	0
		Nagadeepa	Northern	9°37′15″N	79°46′09″E	0
		Point Pedro	Northern	9°50′07″N	80°12′42″E	0
		Silavathurai	Northern	8°40′11″N	79°48′34″E	9–12
NW	Northwestern coast of Sri Lanka	Kalpitiya 1	Northwestern	8°22′33″N	79°45′34″E	2–5
		Kalpitiya 2	Northwestern	8°14′49″N	79°41′19″E	9–13
S	Southern coast of Sri Lanka	Ahangama	Southern	5°57′42″N	80°23′44″E	0.5–2
		Dickwella	Southern	5°57′44″N	80°41′59″E	0
		Godawaya	Southern	6°06′32″N	81°03′13″E	1–5
		Hikkaduwa	Southern	6°07′42″N	80°06′09″E	0.5–3
		Hiriketiya	Southern	5°57′40″N	80°42′32″E	0–4
S	Southern coast of Sri Lanka	Kirinda	Southern	6°12′33″N	81°19′55″E	1–3
		Nilwella	Southern	5°57′42″N	80°43′11″E	1–5
		Polhena	Southern	5°56′03″N	80°31′32″E	1–5
		Rakawa	Southern	6°03′04″N	80°52′01″E	2–5
W	Western coast of Sri Lanka	Beruwala	Western	6°26′58″N	79°58′43″E	0.5–1
		Negombo	Western	7°12′45″N	79°48′52″E	5–8

APPENDIX 2. Locality data recorded in published literature on Sri Lankan regular echinoids. Explanation of symbols: E—eastern coast; GM—Gulf of Mannar; N—northern coast; NW—northwestern coast; S—southern coast; W—western coast.

Site code	Location	Locality name in Literature	Province	Latitude	Longitude	Depth range (m)	Collected by	Recorded by
E	Eastern coast of Sri Lanka	Trincomalee	Eastern					Sarasin & Sarasin (1886, 1887, 1888)
		East coast	Eastern	8°51′N	81°11′E	51	"*Investigator*"	Anderson (1894)
		Trincomalee Harbour	Eastern					Clark (1925)
N	Northern coast of Sri Lanka	Palk straits	Northern			9–37	"*Investigator*"	Anderson (1894)
GM	Gulf of Mannar	East and West Cheval Paars	Northern			13	Herdman	Herdman *et al.* (1904)
		Cheval Paar	Northern			15–16	Herdman	Herdman *et al.* (1904)
		Southwest of Periya Paar	Northern			20–44	Herdman	Herdman *et al.* (1904)
		West of Periya Paar	Northern			66	Herdman	Herdman *et al.* (1904)
		Periya Paar	Northern			16	Herdman	Herdman *et al.* (1904)
		South of Adam's Bridge	Northern			7–73	Herdman	Herdman *et al.* (1904)
		Outside Dutch Modragam Paar	Northern			20–66	Herdman	Herdman *et al.* (1904)
		Arippu/Aripo	Northern				Humbert	Loriol (1874)
NW	Northwestern coast of Sri Lanka	Kalpitiya	Northwestern			0–5	"Sindbad Voyage"	Price & Rowe (1996)
S	Southern coast of Sri Lanka	Welligam Bay	Southern			4–13	Herdman	Herdman *et al.* (1904)
		South of Point de Galle	Southern			29–55	Herdman	Herdman *et al.* (1904)
		Off Galle	Southern			62	Herdman	Herdman *et al.* (1904)
		Station 204	Southern	6°01′N	81°16′E	62		Koehler (1927), Sastry (2007)
		Off S. Ceylon	Southern	6°01′N	81°16′E	60	"*Investigator*"	Mortensen (1943)
		côte Sud de Ceylan	Southern			59		Koehler (1927), Sastry (2007)
		South of Sri Lanka, Station 464	Southern	6°02′30″N	81°29′E	95–124	"*Investigator*"	Sastry (2007)
		Southern coast of Sri Lanka	Southern			260–732		Sastry (2007)
		South of Sri Lanka	Southern	6°01′N	81°16′E	62	"*Investigator*"	Sastry (2007)

……*continued on the next page*

APPENDIX 2. (Continued)

Site code	Location	Locality name in Literature	Province	Latitude	Longitude	Depth range (m)	Collected by	Recorded by
		Ambalangoda	Southern	5°55.5′N	80°56′E	Littoral	F. B. Steiner	California Academy of Sciences (CAS)
		East of Dondra Head	Southern	5°55.5′N	80°56′E	1–5	A. J. Ferreira	California Academy of Sciences (CAS)
		Hiriketiya	Southern	5°57′N	80°43′E			Gayashan & Jayakody (2012)
		Nilwella	Southern	5°57′N	80°42′E			Gayashan & Jayakody (2012)
		Unawatuna, Galle	Southern	5°57′N	80°42′E	0–20	"Sindbad Voyage"	Price & Rowe (1996)
		Galle	Southern			5	"Sindbad Voyage"	Price & Rowe (1996)
W	Western coast of Sri Lanka	Off Colombo	Western			260–732	"*Investigator*"	Anderson (1894)
		West and southwest of Negombo	Western			22–37	Herdman	Herdman *et al.* (1904)
		North of Negombo	Western			15–16	Herdman	Herdman *et al.* (1904)
		Off Kalutara	Western			55	Herdman	Herdman *et al.* (1904)
		Off Mount Lavinia to off Colombo	Western			16–26	Herdman	Herdman *et al.* (1904)
		West coast	Western			46–55	Herdman	Herdman *et al.* (1904)
		Outside Donnan's Paar	Western			37–55	Herdman	Herdman *et al.* (1904)
		Station 204	Western	6°50′20″N	79°36′20″E	329–397		Koehler (1927)
		Station 333	Western	6°31′N	79°38′E	733		Koehler (1927)
		Colombo	Western			260–732		Koehler (1927), Sastry (2007)
			Western	7°02′30″N	79°36′E	836–1077		Koehler (1927)
